# Limited versus Radical Resection in Mitral Valve Infective Endocarditis Surgery

**DOI:** 10.3390/jcdd10040146

**Published:** 2023-03-30

**Authors:** Zaki Haidari, Daniel Wendt, Matthias Thielmann, Heinz Jakob, Arjang Ruhparwar, Mohamed El-Gabry

**Affiliations:** Department of Thoracic and Cardiovascular Surgery, West German Heart and Vascular Centre Essen, University Hospital Essen, Hufelandstrasse 55, 45147 Essen, Germany

**Keywords:** infective endocarditis, mitral valve, cardiac surgery, limited-resection, radical-resection

## Abstract

*Background:* Mitral valve repair is preferred in patients undergoing surgical treatment for infective endocarditis (IE) of the native mitral valve, however, radical resection of infected tissue and patch-plasty might potentially lead to low or non-durable repair. We aimed to compare a limited-resection and non-patch technique with the classic radical-resection technique. *Methods:* Eligible candidates were patients with definitive IE of the native mitral valve undergoing surgery between January 2013 and December 2018. Patients were classified according to the surgical strategy into two groups: limited- versus radical-resection strategy. Propensity score matching was used. Endpoints were repair rate, all-cause mortality (30-day and 2-year), re-endocarditis and reoperation at q-year follow-up. *Results:* After propensity score matching, 90 patients were included. Follow-up was 100% complete. Mitral valve repair rate was 84% in the limited-resection versus 18% in the radical-resection strategy, *p* < 0.001. The 30-day and 2-year mortality were 20% versus 13% (*p* = 0.396) and 33% versus 27% (*p* = 0.490) in the limited-resection versus radical-resection strategy, respectively. The incidence of re-endocarditis during the 2-year follow-up was 4% in the limited-resection strategy versus 9% in the radical-resection strategy, *p* = 0.677. Three patients in the limited-resection strategy underwent reoperation of the mitral valve, while there were none in the radical-resection strategy (*p* = 0.242). *Conclusions:* Although mortality in patients with IE of the native mitral valve remains high, the limited-resection and non-patch surgical strategy is associated with a significantly higher repair rates with comparable 30-day and mid-term mortality, risk of re-endocarditis and re-operation compared to the radical-resection strategy.

## 1. Introduction

Surgical treatment of native mitral valve infective endocarditis (IE) includes the removal of infected tissue and reconstruction of the valvular and sub-valvular structure. Depending on the remaining tissue quality and experience of the surgeon, the native mitral valve is repaired or replaced. Guidelines strongly favor repair over replacement whenever possible, as repair is associated with better outcomes in terms of survival and valve-related events [1,2,3,4,5,6,7,8,9]. Mitral valve repair rates in patients with native mitral valve IE is currently increasing [10] and repair rates of up to 80% have been reported using different patch techniques as a substitute after resection of infected tissue [11,12]. Although the use of patch materials has been successfully used to replace infective valve tissue [13], there are concerns about valve durability and risk of recurrence [14,15,16]. We aimed to compare a non-patch limited resection technique with the classic radical resection and patch technique on repair rates and clinical outcomes in patients with IE of the native mitral valve.

## 2. Patients and Methods

### 2.1. Patients

Eligible candidates for this study were patients with active IE of the native mitral valve undergoing surgical therapy between January 2013 and December 2018. The exclusion criterion was previous mitral valve intervention and/or re-operation. The diagnosis was made according the modified Dukes criteria [17]. All patients were on antibiotic therapy and/or the diagnosis was intraoperatively confirmed based on macroscopic findings of infective endocarditis (e.g., vegetations, infected tissue). Perioperative data were collected from electronic hospital database records. The follow-up was performed by outpatient visits or active telephone follow-up and data on survival and valve-related complications were collected. The institutional ethics committee approved the study, and written informed consent was waived.

### 2.2. Operative Technique

Patients were operated under general anesthesia and orotracheal intubation. Preoperative transesophageal echocardiography was performed to evaluate cardiac and valvular function. Standard aortic and bicaval cannulation techniques were applied. Cardioplegic arrest was achieved by cold crystalloid Bretschneider cardioplegia (Custodiol, Dr. Franz Koehler Chemie, Bensheim, Germany) and topical cooling. After exposure of the mitral valve, a detailed and holistic valve analysis was performed and the presence of infective and concomitant degenerative or rheumatic lesions was identified. The limited-resection technique has been described previously [18]. In short, after removal of the vegetations, macroscopic infected tissue was resected with maximal preservation of the non-infected tissue. The remaining tissue was disinfected with polyvidone and vancomycin. The defects (resections or perforations) were directly closed using interrupted sutures (Cardionyl 5.0, Peters Surgical, Boulogne-Billancourt, France). Other repair techniques included chordal transfer or chordal replacement using artificial polytetrafluoroethylene chordae (W.L. Gore and Association, Inc., Flagstaff, AZ, USA). Finally, the repair was completed with annuloplasty. In the radical-resection strategy, infective lesion resection was extended including a 2 mm resection of the adjacent healthy tissue. Perforations or defects were either directly closed or patch-plasty was performed. In cases with partial leaflet resection, bovine patch-plasty was attempted to replace the defect. In patients with extensive (involvement of both commissures or large anterior mitral leaflet defect) infected tissue, or if the repair did not show a perfect result, mitral valve replacement was performed. In patients with additional cardiac pathology, concomitant procedures were performed accordingly. After weaning from cardiopulmonary bypass, transesophageal echocardiography was performed to evaluate the surgical result.

### 2.3. Postoperative Care

Postoperatively, all patients were transferred to a cardio-surgical intensive care unit. Postoperative care consisted of invasive hemodynamic and pulmonary monitoring and guideline [19,20] directed antibiotic therapy. Transthoracic echocardiography was performed before discharge to evaluate cardiac and valvular function.

### 2.4. Endpoints and Definitions

The primary endpoints were mitral valve repair rate and 30-day all-cause mortality. Secondary endpoints included re-endocarditis, re-operation, and mortality during the two-year follow-up. Re-endocarditis was defined as either relapse (repeat episode of infective endocarditis of the mitral valve caused by the same microorganism as in the index operation) or reinfection (infection caused by a different microorganism, then in the index operation). Re-operation was defined as any reoperation of the mitral valve. Mortality was defined as death by any cause and subdivided into sepsis-related, neurological, cardiac, pulmonary, and others. Sepsis-related mortality was defined as death due to postoperative sepsis and septic shock leading to multiorgan failure.

### 2.5. Statistical Analysis

Data was analyzed using SPSS software version 25 (SPSS Inc., Chicago, IL, USA). Propensity score matching was performed to pair two comparing groups. The matching variables included European System for Cardiac Operative Risk Evaluation (EuroSCORE) II, days between diagnosis and surgery and positive blood culture for staphylococcal species. Matching was performed using 1:1 nearest neighbor with a matching tolerance of 0.1 in the overall propensity score. Continuous variables were expressed as mean or median with standard deviation (SD) or interquartile range (IQR), respectively, and compared using Student’s t-test or the Mann–Whitney test. The Shapiro–Wilk test was used to test for normality. Categorical data were expressed as number of patients and frequencies, and compared using the chi-square test or Fischer’s exact test when appropriate. Kaplan–Meier curves were used to evaluate overall survival and event-free (freedom from re-endocarditis and reoperation) survival rates and the two groups were compared using the log-rank test. A *p*-value < 0.05 was considered statistically significant.

## 3. Results

### 3.1. Baseline Characteristics

Between January 2013 and December 2018, 114 patients with native mitral valve IE underwent surgical therapy (Table S1). After propensity score matching, 90 patients were included into this analysis. Table 1 shows the preoperative baseline characteristics of all patients. There were no statistically significant differences between the two groups in terms of preoperative baseline demographics, clinical status, and the levels of inflammatory parameters. 

The most commonly identified causative pathogen was staphylococcus species in 30 patients, of which 21 patients had *staphylococcus aureus*. Staphylococcus species as the causative microorganism dominated the limited-resection group, while the distribution between staphylococcus and streptococcus species as the causative microorganism was balanced in the radical-resection group. The most common indication for surgery in the limited-resection strategy was large or embolized vegetation, while the distribution between heart failure and large or embolized vegetation was balanced in the radical-resection strategy. However, these differences did not reach statistical significance. The timing of surgery was comparable between the two groups (Table 2).

### 3.2. Operative Characteristics

The operative characteristics of the two groups are listed in Table 3. Isolated mitral valve surgery was performed in 24 patients in the limited-resection group and in 16 patients in the radical-resection group, *p* = 0.09. More patients presented with concomitant procedures in the radical-resection group, however these differences did not reach statistical significance. Cardiopulmonary bypass and aortic cross-clamp times were significantly lower in the limited-resection group.

### 3.3. Endpoints

The endpoints are summarized in Table 4. Mitral valve repair was performed in 38 of the 45 patients in the limited-resection group, resulting in a repair rate of 84%. In the radical-resection group, mitral valve repair was possible in 8 of the 45 patients (repair rate of 18%). The 30-day mortality was 24% in limited-resection group and 13% in the radical-resection group, *p* = 0.178. Postoperative sepsis-related death was the most common cause of mortality in both groups. However, 30-day mortality in the limited-resection group was increased by an additional 5 deaths. Three of the five patients had a preoperative and unknown survival-limiting diagnosis. The causes of death in these patients were brain death due to preoperative stroke, respiratory insufficiency due to preoperatively unknown pulmonary fibrosis, and abdominal bleeding due to preoperatively unknown metastasized carcinoma. 

After the two-year follow-up, all-cause mortality was 33% in the limited-resection group and 26% in the radical-resection group. Two patients with mitral valve repair developed re-endocarditis in the limited-resection group, while four patients (two repaired and two replaced at index operations) had recurrence of mitral valve endocarditis in the radical-resection group, *p* = 0.677. In the limited-resection group, staphylococcus aureus was the causative microorganism in both patients who developed re-endocarditis. The causative microorganisms of endocarditis in the radical-resection group who developed re-endocarditis were variable; among them one had staphylococcus aureus. Three patients underwent re-operation in the limited-resection group, two due to re-endocarditis and one due to severe mitral regurgitation. In the radical-resection group, two patients were not operable (frailty and high-risk) and one patient received successful conservative standard of care therapy.

**Figure 1:**
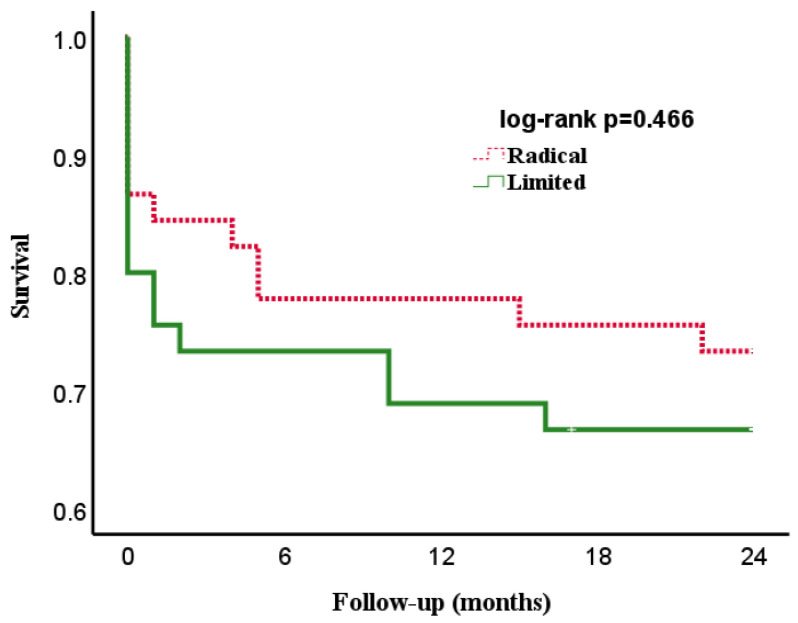
Kaplan–Meier curve showing the overall-survival of patients with infective endocarditis of the native mitral valve undergoing surgical therapy by limited-resection (limited) and radical-resection (radical) strategy.

**Figure 2:**
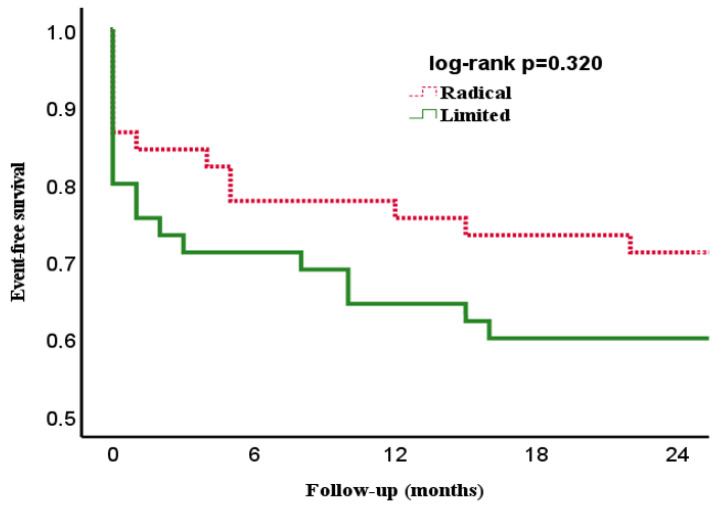
Kaplan–Meier curve showing event-free survival of patients with infective endocarditis of the native mitral valve undergoing surgical therapy by limited-resection (limited) and radical-resection (radical) strategy.

Figure 1 and Figure 2 show the overall- and event-free survival of the two groups during their two-year follow-up. The curves diverge initially due to differences in early mortality and run parallel over the course of follow-up with no statistical differences. 

## 4. Discussion

The current analysis showed the following interesting findings: First, infective endocarditis is associated with significant mortality and morbidity. Second, mitral valve repair could be achieved in a significantly higher proportion in the limited-resection versus the radical-resection group. Third, a limited-resection and non-patch surgical strategy showed comparable outcomes regarding 30-day and mid-term mortality, re-endocarditis, and re-operation. Fourth, the incidence of re-operation over a two-year period did not differ between the two groups. 

Mitral valve repair in patients with active native mitral valve IE is a rather complex procedure but has been shown currently to be associated with improved outcomes [21,22]. To date, mitral valve repair rates treating IE are increasing in experienced and high-volume centers, and these higher repair-rates are mostly achieved by the use of pericardial (bovine) patches to replace the valvular tissue after radical resection. Pericardial patches have been used successfully to augment leaflets in degenerative and rheumatic mitral valve disease in the past [22,23,24]. However, the use of autologous pericardial tissue to replace resected valvular tissue is associated with calcification, tissue retraction, thickening, fibrosis, dehiscence, and loss of pliability in the long run. Furthermore, higher risk of re-endocarditis has been associated with the use of large (>1 cm) pericardial patches [25]. Therefore, the durability of such repair techniques is challenged and meanwhile questioned [14]. We aimed to evaluate the results of limited-resection and non-patch techniques on successful repair rates and outcomes in patients with native mitral valve IE. In this analysis, a higher repair rate could be achieved with the limited-resection method compared to the radical resection technique. Replacement of the mitral valve was performed in the radical resection group once a repair showed an unsatisfactory result, which was needed in more than 80% of cases. The idea of course is, especially in the presence of staphylococcus aureus, not to leave any remnant infective tissue in situ. Our results showed that there were no significant differences regarding staphylococcus regarding the limited versus the radical resection cohort. Furthermore, a lower incidence of valve-related complications and comparable re-intervention and mortality rates were observed when compared to the radical-resection technique over a two-year follow-up period. The incidence of re-endocarditis and/or reoperation during the two-year follow-up showed no statistically significant difference between the two groups (*p* = 0.677). However, three patients in the limited-resection strategy underwent reoperation of the mitral valve, while there were none in the radical-resection strategy (*p* = 0.242). It must be acknowledged however, that three patients in the radical-resection group also had an indication for potential reoperation, but one patient could eventually be managed conservatively with standard of care anti-infective therapy and the remaining patients exhibited far too high a risk for surgery and were deemed inoperable. Overall, although mitral valve repair is associated with improved outcome, we did not observe lower mortality in our population. Early mortality in patients with infective endocarditis is mostly determined by the occurrence of postoperative sepsis and multiorgan failure, which was the case in our cohort. As shown by our two-year follow-up period, the mortality in patients with infective endocarditis remains high. To improve survival, the focus has been on surgical technique (valve repair whenever possible) and reduction of cardiopulmonary bypass and aortic cross-clamp times, which could be achieved in the limited resection group. Despite these improvements, we did not observe any reduction in mortality. Therefore, the focus should be broadened to include patient care in the early postoperative period. Specifically, hemodynamic stability and improvement of organ function should be addressed. 

To date, many new concepts, techniques and anti-infective drugs have been introduced into the treatment of infective endocarditis. For example, the innovative concept of blood purification using hemoadsorption represents one possibility for cytokine removal with the use of sorbents, which has been evaluated recently. Despite not really being a causal treatment of postoperative sepsis or systemic inflammatory response syndrome, the application of adsorptive blood purification techniques, especially in treating infective endocarditis, has gained increased interest [26]. Our group has previously shown that the intraoperative application of hemoadsorption could attenuate the cytokine storm and result in better hemodynamics in the postoperative course [27,28]. Moreover, intraoperative hemoadsorption resulted in a statistically significant difference in sepsis-associated mortality. These results have been duplicated by Kalisnik et al. who also found that hemoadsorption was an independent factor to reduce sepsis-associated mortality [29]. The REMOVE trial failed to show a reduction in the Sequential Organ Failure Assessment score (primary endpoint) in the postoperative course with intraoperative hemoadsorption. However, a significant reduction in cytokines was observed [30]. Recently we were able to show a significant reduction in both the 30- and 90-day mortality in patients with staphylococcus aureus based infective endocarditis [31]. It seems that patient selection plays an important role in the decision making for intraoperative hemoadsorption in patients with infective endocarditis.

## 5. Limitations

In the present analysis, we compared two surgical strategies mainly on mitral valve repair rate in patients with infective endocarditis of the mitral valve. Our study has limitations that should be considered when interpreting the results. First, we need to acknowledge that although this study was not a randomized-controlled trial, both groups were meticulously matched using the propensity-score method. Second, according to the evaluated long-time interval, one could speculate that some treatment algorithms changed over time. Third, the small number of patients included in this ‘pilot’ study does not allow definitive conclusions. Fourth, most of the patients were operated on by two experienced surgeons, resulting in an unavoidable operator’s bias. Nevertheless, to our knowledge, this is the first study to date comparing a limited- versus a radical-resection technique in native mitral valve infective endocarditis patients. However, it should be pointed out that this study does not compare mitral valve repair versus mitral valve replacement in this population. 

## 6. Conclusions

In conclusion, both groups showed comparable outcomes concerning mortality and re-intervention rates. The limited resection method comes with shorter CPB and ACC times and was non-inferior to the radical resection group. Hemoadsorption as an adjunctive therapy might have an impact on better outcomes in such high-risk patients and should be investigated in future studies. 

## Figures and Tables

**Table 1:** Baseline characteristics.

Variables	Limited-Resection n = 45	Radical-Resection n = 45	*p*
**Demographics**			
Age, *years ± SD*	60 ± 14	62 ± 13	0.446
Gender, female, *n* (%)	15 (33)	16 (36)	0.824
Diabetes, *n* (%)	10 (22)	12 (27)	0.624
Drug abuse, *n* (%)	3 (7)	1 (2)	0.616
Previous stroke, *n* (%)	18 (40)	16 (36)	0.664
- Endocarditis related, *n*	17	15	0.660
- Neurologic dysfunction, *n*	13	12	0.814
CAD, *n* (%)	10 (22)	11 (24)	0.803
Atrial fibrillation, *n* (%)	12 (27)	13 (29)	0.814
Pulmonary disease, *n* (%)	6 (13)	6 (13)	>0.999
Dialysis, *n* (%)	4 (9)	7 (16)	0.334
Liver disease, *n* (%)	4 (9)	2 (4)	0.677
PVD, *n* (%)	6 (13)	7 (16)	0.764
Previous CABG, *n* (%)	3 (7)	3 (7)	>0.999
Previous PCI, *n* (%)	2 (4)	3 (7)	>0.999
Previous valve surgery, *n* (%)	3 (7)	4 (9)	>0.999
EuroSCORE II, *mean ± SEM*	10 ± 2	11 ± 2	0.642
**Clinical status**			
NYHA fc III-IV, *n* (%)	25 (56)	21 (47)	0.399
Intubated, *n* (%)	5 (11)	4 (9)	0.725
Vasopressor need, *n (%)*	5 (11)	5 (11)	>0.999
Surgical delay, *days ± SD*	16 ± 2	17 ± 3	0.940
**Inflammatory status**			
CRP, *mg/dL (IQR)*	4.90 (1.95–10.25)	5.00 (1.95–8.55)	0.692
PCT, *ng/mL (IQR)*	0.21 (0.08–0.36)	0.28 (0.14–0.61)	0.199
WBC, *10^9^/L (IQR)*	8.24 (5.56–11.52)	8.60 (6.56–12.27)	0.169
**Echocardiographic parameters**			
LVEF > 50%, *n* (%)	35 (78)	39 (87)	0.270
Severe MR, *n* (%)	24 (53)	33 (73)	0.049
Concomitant AV IE, *n* (%)	8	12	0.310
Concomitant TV IE, *n* (%)	3	2	>0.999

Data are presented as mean ± SD, median (interquartile range) or number (percentage); BMI, body mass index; CAD, coronary artery disease; PVD, peripheral artery disease; CABG, coronary artery bypass grafting; PCI, percutaneous coronary intervention; EuroSCORE, European System for Cardiac Operative Risk Evaluation; NYHA fc, New York Heart Association functional classification; CRP, c-reactive protein; PCT, procalcitonin; WBC, white blood count; LVEF, left ventricular ejection fraction; MR, mitral regurgitation; AV, aortic valve; TV, tricuspid valve; IE, infective endocarditis. SD, standard deviation; IQR, interquartile range.

**Table 2:** Causative microorganism and surgical indication and timing.

Variables	Limited-Resection n = 45	Radical-Resection n = 45	*p*
**Causative microorganism**			
Staphylococcus species, *n* (%)	19 (42)	11 (24)	0.074
- Staphylococcus aureus, *n*	13	8	0.213
Streptococcus species, *n* (%)	5 (11)	12 (27)	0.059
Enterococcus species, *n* (%)	5 (11)	6 (13)	0.748
Others, *n* (%)	4 (9)	4 (9)	>0.999
Negative, *n* (%)	12 (27)	12 (27)	>0.999
**Indication for surgery**			
Heart failure, *n* (%)	8 (18)	16 (36)	0.057
Large or embolized vegetation, *n* (%)	28 (62)	19 (42)	0.058
Uncontrolled infection, *n* (%)	8 (18)	8 (18)	>0.999
Valvular disease, *n* (%)	1 (2)	2 (4)	>0.999
**Timing of surgery**			
Elective (>7 days), *n* (%)	34 (76)	31 (69)	0.480
Urgent (2–7 days), *n* (%)	7 (15)	8 (18)	0.777
Emergent (<2 days), *n* (%)	4 (9)	6 (13)	0.502

Data are presented as number (percentage).

**Table 3:** Operative characteristics.

Variables	Limited-Resection n = 45	Radical-Resection n = 45	*p*
Isolated mitral valve surgery, *n* (%)	24 (53)	16 (36)	0.090
Concomitant CABG, *n* (%)	4 (9)	7 (16)	0.334
Concomitant AV surgery, *n* (%)	13 (29)	15 (33)	0.649
Concomitant TV surgery, *n* (%)	5 (11)	7 (16)	0.535
Minimally invasive, *n* (%)	2 (4)	6 (13)	0.266
CPB time, *minutes (IQR)*	92 (73–117)	128 (111–172)	<0.001
ACC time, *minutes (IQR)*	59 (45–78)	84 (73–121)	<0.001

Data are presented as number (percentage) or median (interquartile range); CABG, coronary artery bypass grafting; CABG, coronary artery bypass graft; AV, aortic valve; TV, tricuspid valve; CPB, cardiopulmonary bypass; ACC, aortic cross-clamp; IQR, interquartile range.

**Table 4:** Endpoints.

Variables	Limited-Resection n = 45	Radical-Resection n = 45	*p*
**Primary**			
Repair rate, *n* (%)	38 (84)	8 (18)	<0.001
Mortality (30-Day), *n* (%)	11 (24)	6 (13)	0.178
- Sepsis-related, *n*	6	6	>0.999
**Secondary**			
Mortality (2-Year), *n* (%)	15 (33)	12 (26)	0.490
Re-endocarditis, *n* (%)	2 (4)	4 (9)	0.677
Re-operation, *n* (%)	3 (7)	-	0.242
Postoperative IABP, *n* (%)	2 (4)	2 (4)	>0.999
Postoperative ECMO, *n* (%)	-	2 (4)	0.494
Dialysis, *n* (%)	9 (20)	11 (24)	0.612
Reintubation, *n* (%)	5 (11)	13 (29)	0.035
ICU-stay, *days (IQR)*	5 (2–10)	6 (4–11)	0.129
Hospital stay, *days (IQR)*	13 (10–21)	14 (9–20)	0.771

Data are presented as number (percentage) or median (interquartile range); IABP, intra-aortic balloon pump; ECMO, extracorporeal membrane oxygenation; ICU, intensive care unit.

## Data Availability

The data presented in this study are available on request from the corresponding author. The data are not publicly available due to privacy restrictions.

## Supplementary Materials

The following supporting information can be downloaded at: https://www.mdpi.com/article/10.3390/jcdd10040146/s1, Table S1: Baseline characteristics of the unmatched (n = 114) population.
