# Supplementary material for: Limited versus Radical Resection in Mitral Valve Infective Endocarditis Surgery
Source: J Cardiovasc Dev Dis. 2023 Mar 30;10(4):146. doi: 10.3390/jcdd10040146 (PMC10144351; doi:10.3390/jcdd10040146)
Supplement: Supplementary file 1 [file jcdd-10-00146-s001.zip › jcdd-2218735-supplementary.pdf]

Supplementary Table

Table S1. Baseline characteristics of the unmatched (n=114) population.

**Variables**	**Limited-resection n=63**	**Radical-resection n=51**	***p***
**Demographics**			
Age, *years±SD*	60±13	62±13	0.437
Gender, female, *n (%)*	22 (35)	18 (35)	0.967
Diabetes, *n (%)*	11 (17)	14 (27)	0.200
Drug abuse, *n (%)*	5 (8)	2 (4)	0.475
Previous stroke, *n (%)*	27 (43)	18 (35)	0.411
- endocarditis related, n (%)	25 (40)	17 (33)	0.485
- neurologic dysfunction, n (%)	20 (32)	14 (27)	0.618
CAD, *n (%)*	13 (21)	14 (27)	0.395
Atrial fibrillation, *n (%)*	15 (24)	14 (27)	0.657
Pulmonary disease, *n (%)*	7 (11)	7 (14)	0.672
Dialysis, *n (%)*	5 (8)	8 (16)	0.196
Liver disease, *n (%)*	5 (8)	3 (6)	0.730
PVD, *n (%)*	7 (11)	10 (20)	0.205
Previous CABG, *n (%)*	3 (5)	3 (6)	>0.999
Previous PCI, *n (%)*	3 (5)	3 (6)	>0.99
Previous valve surgery, *n (%)*	3 (5)	6 (12)	0.295
EuroSCORE II, *mean±SEM*	8.03±1.35	15.16±2.20	0.007
**Clinical status**			
NYHA fc III-IV, *n (%)*	29 (56)	27 (53)	0.463
Intubated, *n (%)*	5 (8)	8 (16)	0.196
Vasopressor need, n *(%)*	5 (8)	9 (18)	0.116
Surgical delay, days±SD	15±12	15±17	0.989
**Inflammatory status**			
CRP, mg/dL (IQR)	4.5 (1.8-9.8)	6.9 (2.2-10.0)	0.429
PCT, ng/ml (IQR)	0.17 (0.05-0.32)	0.42 (0.16-0-82)	0.071
WBC, 10^9/L (IQR)	8.35 (6.27-11.75)	9.83 (7.47-13.75)	0.235
**Echocardiographic parameters**			
LVEF >50%, *n (%)*	53 (84)	43 (84)	0.978
Severe MR, *n (%)*	37 (59)	37 (73)	0.124
Concomitant AV IE, *n (%)*	10 (16)	13 (25)	0.203
Concomitant TV IE, *n (%)*	7 (11)	2 (4)	0.184

Data are presented as mean±SD, median (interquartile range) or number (percentage); BMI, body mass index; CAD, coronary artery disease; PVD, peripheral artery disease; CABG, coronary artery bypass grafting; PCI, percutaneous coronary intervention; EuroSCORE, European System for Cardiac Operative Risk Evaluation; NYHA fc, New York Heart Association functional classification; CRP, c-reactive protein; PCT, procalcitonin; WBC, white blood count; LVEF, left ventricular ejection fraction; MR, mitral regurgitation; AV, aortic valve; TV, tricuspid valve; IE, infective endocarditis. SD, standard deviation; IQR, interquartile range.
